# Transformation of Rasch model logits for enhanced interpretability

**DOI:** 10.1186/s12874-022-01816-1

**Published:** 2022-12-23

**Authors:** Joakim Ekstrand, Albert Westergren, Kristofer Årestedt, Amanda Hellström, Peter Hagell

**Affiliations:** 1 The PRO-CARE Group, Faculty of Health Sciences, Kristianstad University, SE-291 88, Kristianstad, Sweden; 2 Faculty of Health and Life Sciences, Linnaeus University, Kalmar, Sweden; 3 Department of Research, Region Kalmar County, Kalmar, Sweden

**Keywords:** Measurement, Outcome measurement, Rasch model, Rating scales, Software, Transformation, Log-odds units

## Abstract

**Background:**

The Rasch model allows for linear measurement based on ordinal item responses from rating scales commonly used to assess health outcomes. Such linear measures may be inconvenient since they are expressed as log-odds units (logits) that differ from scores that users may be familiar with. It can therefore be desirable to transform logits into more user-friendly ranges that preserve their linear properties. In addition to user-defined ranges, three general transformations have been described in the literature: the least measurable difference (LMD), the standard error of measurement (SEM) and the least significant difference (LSD). The LMD represents the smallest possible meaningful unit, SEM relates the transformed scale values to measurement uncertainty (one unit on the transformed scale represents roughly one standard error), and LSD represents a lower bound for how coarse the transformed scale can be without loss of valid information. However, while logit transformations are relatively common in the health sciences, use of LMD, SEM and LSD transformations appear to be uncommon despite their potential role.

**Methods:**

Logit transformations were empirically illustrated based on 1053 responses to the Epworth Sleepiness Scale. Logit measures were transformed according to the LMD, SEM and LSD, and into 0–10, 0-100, and the original raw score (0–24) ranges. These transformations were conducted using a freely available Excel tool, developed by the authors, that transforms logits into user-defined ranges along with the LMD, SEM and LSD transformations.

**Results:**

Resulting LMD, SEM and LSD transformations ranged 0-34, 0-17 and 0-12, respectively. When considering these relative to the three user-defined ranges, it is seen that the 0-10 range is narrower than the LSD range (i.e., loss of valid information), and a 0-100 range gives the impression of better precision than there is, since it is considerably wider than the LMD range. However, the 0-24 transformation appears reasonable since it is wider than the LSD, but narrower than the LMD ranges.

**Conclusions:**

It is suggested that LMD, SEM and LSD transformations are valuable for benchmarking in deciding appropriate ranges when transforming logit measures. This process can be aided by the Excel tool presented and illustrated in this paper.

**Supplementary Information:**

The online version contains supplementary material available at 10.1186/s12874-022-01816-1.

## Background

Rating scales are commonly used as a means of quantifying latent variables across a variety of fields, such as education, psychology, economics, and the health sciences. For example, in clinical studies it is common to use rating scale-based instruments (e.g., questionnaires and observational assessments) to determine interventional outcomes such as symptom severity, activity performance and self-reported health. This typically involves a process of summing individual item scores into total scores that are treated as measures of the variable that the instrument intends to represent [1]. However, just as individual item scores, raw total scores are ordinal. This means that it is not possible to make inferences regarding what magnitude a certain change or difference in scores represents, which in turn prohibits sound comparisons. Furthermore, the ordinal nature of raw total scores means that they are unsuitable for common calculations such as the mean and other parametric statistics [2, 3].

To overcome issues related to the use of raw scores and to improve the quality assurance of rating scale-based instruments, the Rasch measurement model is recommended over traditional psychometric methods [1, 4]. In addition to being a powerful means to disclose and diagnose anomalies in the measurement process, the Rasch model allows for linear measurement to be accomplished based on ordinal item responses. However, these linear measures, expressed as log-odds units (logits), may be inconvenient for practical use and interpretation since they range from negative to positive values that typically are reported with two or three decimals [5, 6]. Such values differ from the non-negative integer raw scores that users may be familiar with. It can therefore be desirable to transform linear logit locations into more user-friendly ranges that can be used in further applications of the instrument [7].

In this paper, different approaches to transform Rasch model derived logits into more user-friendly ranges that preserve their linear properties are described. We then use empirical data to illustrate and discuss these transformations, and an Excel tool to aid researchers and practitioners in conducting logit transformations is proposed.

### The Rasch model

The Rasch measurement model [8] can be used to overcome challenges associated with the use of raw total scores, and to assure that rating scale-based instruments are of acceptable standard and appropriate for their intended use [9–11]. The Rasch model assumes unidimensionality and local response independence, which both are assessed by various tests of fit of the data to the model as a means of quality control. When data exhibits sufficient fit to the model, linear measurement is accomplished. While issues related to fit are central in Rasch measurement, this will not be discussed here, but readers are instead referred to other sources [e.g., 9, 12, 13].

The Rasch model estimates the locations of both items and persons on a common latent continuum from less to more of the measured variable. The unit used to locate (or measure) persons and items along the latent continuum is the logit (or log-odds unit) that, in the case of dichotomous item responses, is derived from the natural logarithm (*ln*) of the probability of scoring 1 over the probability of scoring 0. The resulting logit represents the difference between the location (e.g., ability) of the person and the location (e.g., difficulty) of the item. Formally, the basic Rasch model for dichotomous item response data takes the form

1 $$ln\left(\frac{{P}_{ni1}}{1-{P}_{ni1}}\right)={\beta }_{n}-{\delta }_{i},$$

where *P*_*ni1*_ is the probability of person *n* scoring 1 (rather than 0) on item *i*, *β*_*n*_ is the location of person *n*, and *δ*_*i*_ is the location of item *i*. The model may also be expressed as

2 $${P}_{ni1}=\frac{{exp}^{\left({\beta }_{n}-{\delta }_{i}\right)}}{1+{exp}^{\left({\beta }_{n}-{\delta }_{i}\right)}}.$$

One distinguishing feature of the model is that it yields separate person and item locations that are independent of each other [9]. In addition, the model estimates the precision (standard error, SE) of these locations. The SE is also expressed on the logit scale and provides direct information on the measurement uncertainty associated with individual person and item locations. The SE of measurement is not constant but vary along the measurement continuum. For person locations, it is greater for people with very low and high scores. The location of persons who score the minimum or maximum possible score on an instrument cannot be estimated since their levels on the variable are below or above the levels that the instrument represents; their locations and associated SEs are infinite [9, 14]. However, Rasch model estimation software typically set the total scores of such persons at non-minimum and non-maximum values so that persons with extreme scores can be included rather than excluded in further analyses. For example, the Rasch Unidimensional Measurement Model 2030 (RUMM2030) software derives these extrapolated extreme locations (and associated SEs) using a geometric mean algorithm involving the three lowest and highest item location estimates for the items attempted by persons with minimum or maximum possible scores, respectively [9].

For completeness it should also be noted that the Rasch model can be generalized and expressed as the polytomous Rasch model, which is applicable when items have more than two ordered response categories [13, 15–18]. There are different versions of the polytomous Rasch model, often referred to as the rating scale and partial credit model, respectively [13]. While these variations are mathematically equivalent [17], the rating scale model assumes that response categories are the same and function the same way across all items, whereas the partial credit model does not [13]. The polytomous Rasch model takes the following general form:

3 $${P}_{nix}=\frac{{exp}^{-{\tau }_{1i}-{\tau }_{2i}\dots -{\tau }_{xi}+x\left({\beta }_{n}-{\delta }_{i}\right)}}{{\sum }_{{x}^{{\prime }}=0}^{{m}_{i}}{exp}^{-{\tau }_{1i}-{\tau }_{2i}\dots -{\tau }_{{x}^{{\prime }}i}+{x}^{{\prime }}\left({\beta }_{n}-{\delta }_{i}\right)}},$$

where *P*_*nix*_ is the probability of person *n* to score *x* on item *i*, *τ*_*xi*_ (*x* = 1, 2, …*m*_*i*_) are the thresholds that divide the latent continuum of item *i* into *m*_*i*_+1 ordered categories, and *x* is the observed item score.

As summarized above, given acceptable model fit, the Rasch model enables linearization of raw total scores into interval logit measures with known measurement uncertainties that are appropriate for, e.g., parametric statistics and comparisons of magnitudes [6, 10, 11, 19]. The interval nature of the logit scale means that its origin is unspecified, but the mean item location is typically set to zero and used as an arbitrary origin, where higher and lower raw total scores are represented by higher (positive) and lower (negative) logit values, respectively. However, the occurrence of both positive and negative values that typically are reported to the second or third decimal can be confusing and abstract, particularly to practitioners and researchers that are used to interpret a certain non-negative integer raw total score range.

To facilitate interpretation and use of logits, it is therefore often desirable to transform these into a more user-friendly range that preserves the linear properties without unnecessary loss of information or precision [20]. In some cases, for example with established instruments, it may be desirable to transform the logit into the original integer raw total score range. In other situations, a 0–10 or a 0-100 (or any other user defined) range may be sought. Although it is possible to transform the logit based on any analysis according to the Rasch model, there are some considerations that should be pointed out. Arguably, the purpose of transforming the logit primarily lies in facilitating future use of an instrument in a way that preserves linearity and yields valid estimates of measurement uncertainty of individual person scores without the need to apply the Rasch model. For the transformed scale (as well as the original logit locations) to be generalizable and useful in a wider context (not only for the data at hand) it is therefore recommended that the transformation is based on complete item response data from an appropriate sample that is representative for the instrument’s intended target population. To gain generalizability, estimated locations (of persons, items and response category thresholds) and associated SEs should also be as stable as possible, which is achieved from well-targeted and relatively large samples using the final version of the instrument. For example, malfunctioning response categories or differential item functioning that compromises invariant measurement should have been rectified earlier during the instrument development or revision process.

### Transforming the logit

Transformations of the logit is relatively straight forward since they are linear, and the actual procedure and mathematics in doing so were first described by Wright and Stone [14] and later by, e.g., Smith [21] and Smith, Jr. [20]. The basic logit transformation formula is

4 $$y=m+sx,$$

where *y* is the new transformed value, *m* is the location factor (= wanted minimum – current logit minimum × *s*), *s* is the spacing factor (= wanted new range / current logit range), and *x* is the logit measure. The spacing factor preserves the relative size of the intervals between logit measures, and the location factor realigns the scale to a new wanted minimum. As seen in Eq. 4, logit measures may be transformed into any desired new score range, and the defining factor in the transformation is the spacing factor.

In addition to transforming the logit locations, the associated SEs also need to be transformed to provide information on the measurement uncertainty on the new transformed scale. This is achieved by multiplying the spacing factor used in the transformation of the logit locations by the original logit SE. That is,

5 $${SE}_{y}=s\left({SE}_{x}\right),$$

where *SE*_*y*_ is the new transformed SE, *s* is the spacing factor and *SE*_*x*_ is the original logit SE.

As described earlier [14, 20], there are some considerations that need to be made when conducting logit transformations. Specifically, one needs to consider what range to transform into and whether this range is reasonable from a measurement perspective. For example, if transformed into an integer range that is too wide there is a risk that the transformed scores give the impression of a level of precision that appears better than it is and there will be wide gaps between achievable scores. Conversely, if transformed into a range that is too narrow, precision and information may be lost. Wright and Stone [14] outline different transformations with different properties related to such considerations. Again, the key in these different transformations is the definition of the spacing factor *s*, and Wright and Stone [14] suggest three alternatives: the least measurable difference (LMD), the standard error of measurement (SEM) and the least significant difference (LSD). These three transformations are summarized below, while details regarding, e.g., their derivations are available elsewhere [14].

The LMD stems from the least observable difference (i.e., one raw score point) and estimates the smallest possible meaningful unit. Therefore, the LMD defines a spacing factor so that the logit LMD represents at least one integer on the new transformed measure. Wright and Stone [14] suggests 6/L as a working definition of the LMD, implying an LMD spacing factor (*s*_*LMD*_) of

6 $${s}_{LMD}= \frac{1}{6/L}= \frac{L}{6},$$

where *L* is the maximum possible raw total score when the minimum score is set at 0. However, Wright and Stone [14] point out that there may be cases where a combination of characteristics of the instrument as well as of the persons means that a spacing factor up to L/4 may be needed to guarantee that each logit location for the raw total scores is transformed into unique integers in the new scale. On the other hand, in some cases a factor down to L/9 may be sufficient to guarantee unique integers. However, an LMD spacing factor of L/6 is recommended unless it renders non-unique integers in the new scale, in which case a redefined LMD spacing factor may be considered.

The SEM based spacing factor relates the transformed scale values to measurement uncertainty. This has an advantage in terms of interpretation since one unit on the new transformed SEM based measure represents roughly one SE, and hence +/- two units represents the approximate 95% confidence interval (CI). However, an obvious disadvantage is that it is somewhat less discriminating than a scale based on the LMD. Wright and Stone [14] relate the SEM to the LMD (SEM = √LMD) and suggest 2.5/√L as a working value for SEM, resulting in a spacing factor (*s*_*SEM*_) of

7 $${s}_{SEM}= \frac{1}{2.5/\sqrt{L}}= \frac{\sqrt{L}}{2.5} .$$

However, it should be noted that it is common for instruments to measure with lower precision towards the ends of their ranges, which results in larger SEs for the logit positions towards the ends. Therefore, the principle that one step on the new SEM-based integer scale corresponds to one SE will not necessarily be true for the entire range of the scale, with the ends of the scale being the most common exceptions.

Finally, the LSD represents an estimated lower bound on how coarse the new transformed scale can be without loss of valid information. The working value of LSD has been suggested to be 3.5/√L [14], which corresponds to 1.4 SEM and yields a minimum spacing factor (*S*_*LSD*_) of

8 $${s}_{LSD}= \frac{1}{3.5/\sqrt{L}}= \frac{\sqrt{L}}{3.5}.$$

Equations 6, 7 and 8 yields that these spacing factors are ordered relative to one another according to *s*_*LMD*_ > *s*_*SEM*_ > *s*_*LSD*_, provided that the raw total score range is more than 6 (i.e., L > 6).

Other approaches include transformation of the linear logit measures into the same range as that of the original raw total score or other ranges, for example 0–10 or 0-100. Indeed, this is a common approach in the health sciences [7, 22–28]. However, regardless of which transformation that is considered, we suggest that the properties of the LMD, SEM and LSD transformations make them useful as a means of quality control and benchmarking in deciding on the most appropriate transformation. If, for example, the new transformed measure has a range that exceeds that from the LMD, this would suggest that the transformed scores give the impression of a level of discrimination that goes beyond their actual precision. Conversely, if the range of the new transformed measure is less than that of the LSD, information will be lost because the transformation is too coarse. However, we are unaware of any studies in the health sciences that have taken advantage of and accounted for the properties of the LMD, SEM and LSD spacing factors in their logit transformations.

Next, we present an empirical illustration of the transformations outlined above and how the LMD, SEM and LSD can be used to enhance interpretation and serve as sources of benchmarking user-defined transformations.

## Methods

We used complete item response data to the Epworth Sleepiness Scale (ESS) from 1053 people (52% women) from a random sample of people between 20 and 64 years of age (mean, 43.3; SD, 12.2) drawn from the population register of an averaged sized mid-Swedish municipality [29]. The original study was a survey related to sleep and sleepiness and included the ESS in addition to demographic and other sleep related questionnaires [29]. The ESS is an 8-item instrument commonly used in sleep research that concerns the propensity of dozing off or falling asleep during various day-to-day activities [30]. Each item has four ordered response categories, ranging between ‘Would never doze’ and ‘High chance of dozing’ (scored 0–3). The eight item scores are summed into a raw total score that can range between 0 and 24 (24 = more daytime sleepiness); scores > 10 suggest abnormal levels of daytime sleepiness [31]. ESS item response data were analysed according to the polytomous Rasch model (partial credit model) using RUMM2030 Professional Edition (version 5.4; RUMM Laboratory Pty Ltd., Perth, WA, Australia). Linear transformations of person logit locations were made into 0–24 (the original raw score range), 0–10 and 0-100 ranges, as well as by using the LMD, SEM and LSD based spacing factors using Microsoft Excel for Microsoft 365 (version 2208, Build 16). All transformations were made into integers.

## Results

**Fig. 1:**
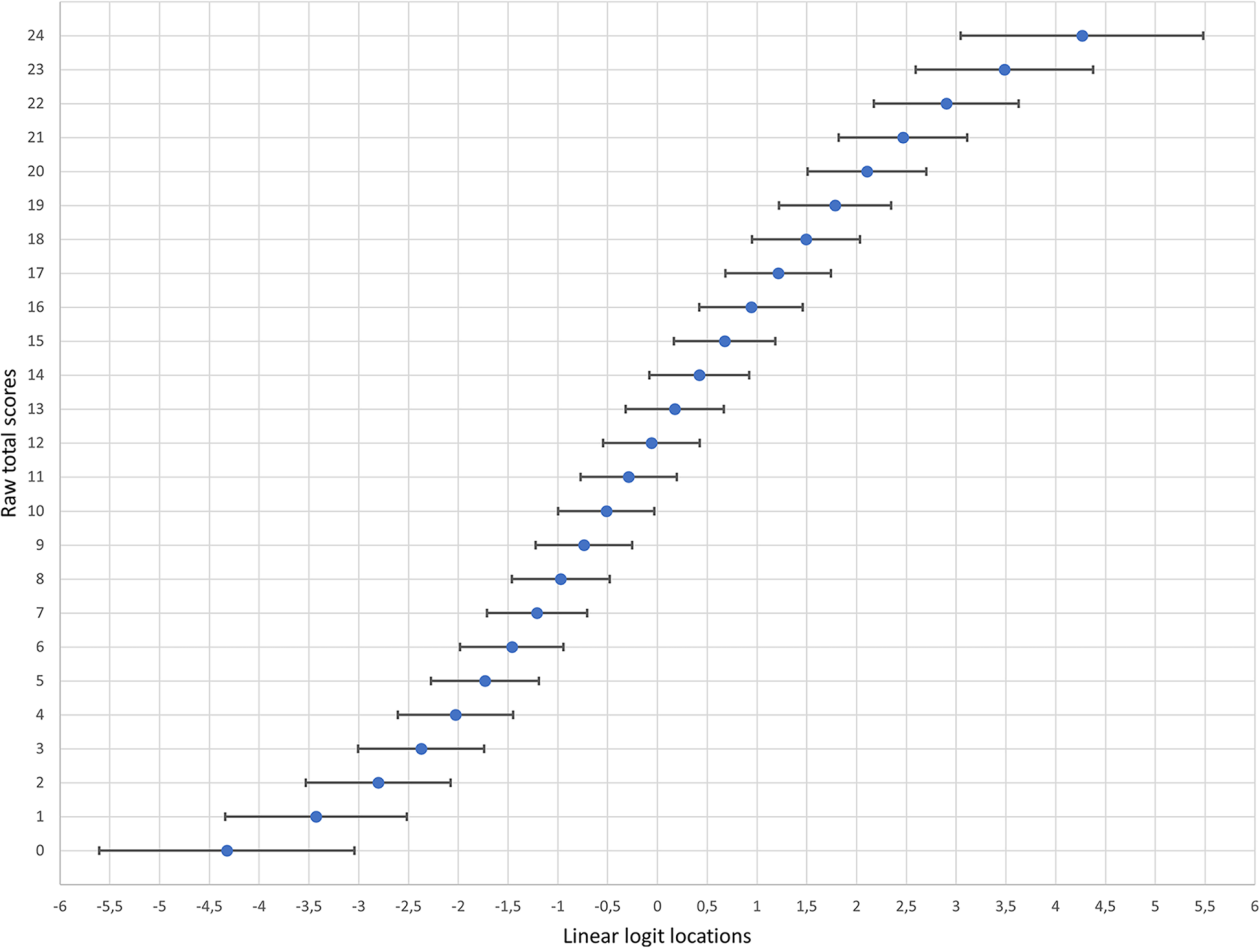
The relationship between ordinal raw total scores (y-axis) of the Epworth Sleepiness Scale (ESS) and their corresponding linearised logit locations (x-axis). Error bars represent standard errors for each logit location

Figure 1 illustrates the relationship between ordinal raw total ESS scores and their accompanying linear logit measures together with the logit SEs at each corresponding raw score point.

Corresponding numerical data are presented in Table 1 (columns A and B). In addition, Table 1 exhibits the resulting logit transformations into the 0–24 original raw score (column C), 0–10 (column D) and 0-100 (column E) ranges, as well as transformations based on the LMD (column F), SEM (column G) and LSD (column H) spacing factors.

**Table 1:** Raw total ESS scores, linear logit locations and transformations to different score ranges

Columns:														
A	B		C		D		E		F		G		H	
			Linear logit transformations^a^											
			0-24 range (*s* = 2.79)		0-10 range (*s* = 1.16)		0-100 range (*s* = 11.64)		LMD (*s* = 4.00)		SEM (*s* = 1.96)		LSD (*s* = 1.40)	
Raw total score	Logit location	Logit SE	New location	New SE	New location	New SE	New location	New SE	New location	New SE	New location	New SE	New location	New SE
0^b^	-4.323	1.28	0	3.6	0	1.5	0	14.9	0	5.1	0	2.5	0	1.8
1	-3.428	0.91	3	2.5	1	1.1	10	10.6	4	3.6	2	1.8	1	1.3
2	-2.805	0.73	4	2.0	2	0.8	18	8.5	6	2.9	3	1.4	2	1.0
3	-2.373	0.63	5	1.8	2	0.7	23	7.4	8	2.5	4	1.2	3	0.9
4	-2.029	0.58	6	1.6	3	0.7	27	6.7	9	2.3	4	1.1	3	0.8
5	-1.732	0.54	7	1.5	3	0.6	30	6.3	10	2.2	5	1.1	4	0.8
6	-1.462	0.52	8	1.5	3	0.6	33	6.0	11	2.1	6	1.0	4	0.7
7	-1.210	0.50	9	1.4	4	0.6	36	5.9	12	2.0	6	1.0	4	0.7
8	-0.971	0.49	9	1.4	4	0.6	39	5.7	13	2.0	7	1.0	5	0.7
9	-0.740	0.49	10	1.4	4	0.6	42	5.6	14	1.9	7	1.0	5	0.7
10	-0.514	0.48	11	1.3	4	0.6	44	5.6	15	1.9	7	0.9	5	0.7
11	-0.289	0.48	11	1.3	5	0.6	47	5.6	16	1.9	8	0.9	6	0.7
12	-0.060	0.49	12	1.4	5	0.6	50	5.7	17	1.9	8	1.0	6	0.7
13	0.175	0.49	13	1.4	5	0.6	52	5.7	18	2.0	9	1.0	6	0.7
14	0.420	0.50	13	1.4	6	0.6	55	5.8	19	2.0	9	1.0	7	0.7
15	0.676	0.51	14	1.4	6	0.6	58	5.9	20	2.0	10	1.0	7	0.7
16	0.941	0.52	15	1.5	6	0.6	61	6.0	21	2.1	10	1.0	7	0.7
17	1.213	0.53	15	1.5	6	0.6	64	6.2	22	2.1	11	1.0	8	0.7
18	1.492	0.54	16	1.5	7	0.6	68	6.3	23	2.2	11	1.1	8	0.8
19	1.785	0.56	17	1.6	7	0.7	71	6.6	24	2.3	12	1.1	9	0.8
20	2.103	0.60	18	1.7	7	0.7	75	6.9	26	2.4	13	1.2	9	0.8
21	2.466	0.65	19	1.8	8	0.8	79	7.5	27	2.6	13	1.3	10	0.9
22	2.902	0.73	20	2.0	8	0.8	84	8.5	29	2.9	14	1.4	10	1.0
23	3.485	0.89	22	2.5	9	1.0	91	10.4	31	3.6	15	1.7	11	1.2
24^c^	4.265	1.22	24	3.4	10	1.4	100	14.2	34	4.9	17	2.4	12	1.7

*ESS* Epworth Sleepiness Scale, *SE* standard error, *s* spacing factor, *LMD* least measurable difference, *SEM* standard error of measurement, *LSD* least significant difference

^a^See Equations 4-8 for transformation formulae

^b^Estimated based on a hypothetical raw score of 0.185 (RUMM2030 Professional Edition, version 5.4)

^c^Estimated based on a hypothetical raw score of 23.795 (RUMM2030 Professional Edition, version 5.4)

**Fig. 2:**
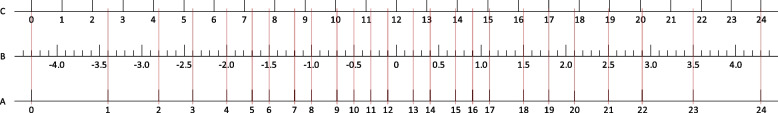
Red vertical lines illustrate the relationship between ordinal raw total Epworth Sleepiness Scale (ESS) scores (**A**), corresponding Rasch model derived person logit locations (**B**), and linear logit transformation into the original 0–24 range without rounding into integer scores (**C**)

The transformations presented in Table 1 can be used to assess and guide the choice of transformation in relation to the properties of the transformations in combination with considerations related to the intended use of the scale. The data presented in Table 1 show that the linear logit transformation into an integer 0–24 range (column C) is narrower than that based on the LMD (range 0–34; column F). This means that the 0–24 transformation is reasonable in the sense that it does not yield a discrimination that gives the impression of a level of precision that appears better than it is. It is also wider than that achieved with the LSD spacing factor (range 0–12; column H). In contrast, the 0–10 range transformation (column D) is narrower than the 0–12 LSD based range (column H). Although the 0–10 range could be conceived, this would be at the expense of information loss since this range is narrower than that achieved with the LSD spacing factor (column H). Conversely, transformation into a 0-100 range would yield the impression of a higher level of precision than there is, since the LMD based transformation only ranges from 0 to 34 (column F), which represents the smallest possible meaningful units. Although the 0-100 range does not represent more than the original 25 possible scale levels, there is a risk that the implied finer precision affects interpretations. It is therefore argued that there is an advantage if the transformed range do not considerably exceed that of LMD derived ranges. In the current ESS example, the widest reasonable transformed range would be 0–34 (column F). Disregarding the transformed 0-100 range (column E), this is illustrated by the fact that the LMD based spacing factor is the only transformation where each original raw score is represented by a unique integer value. Figure 2 illustrates the relationship between the ESS raw scores, their corresponding logit locations, and a linear logit transformation back to the original 0–24 range.

As illustrated in Figs. 1 and 2, there is not a linear relationship between raw total scores and linear logit measures. Distances between raw score points are wider towards the upper and lower ends of the scale and narrower in the middle of the range, where they also approach linearity; a pattern that is representative for virtually all scales [6, 7, 9]. This illustrates that linearization of ordinal raw total scores is relevant for the quantification of changes and differences between individuals. The relationship between ordinal and linearized scores is also made explicit by the logit transformations (Table 1). For example, consider ordinal raw scores of 0 and 2 (column A), a 2-point difference. In the linear 0–24 range transformation (column C) raw scores of 0 and 2 are represented by 0 and 4, a 4-point difference. Considering the same 2-point difference change near the centre of the ordinal scale (column A), say scores of 10 and 12, it is seen that this translates to transformed linear scores of 11 and 12 points (column C), a 1-point difference. This is also illustrated in Fig. 2 A and B, but here the transformation is not rounded into integers (Fig. 2 C). For example, an ordinal raw total ESS score of 2 (Fig. 2 A) is represented by a linear logit location of about − 2.8 (Fig. 2B) that in turn represents a score of about 4.2 following linear transformation into the original 0–24 range (Fig. 2 C). It should be noted that in Table 1 this value is rounded to 4 in order to retain integer scores; a “loss” of precision that is clearly acceptable given the measurement uncertainties (SEs) associated with each score point. For comparison, it is also seen that the corresponding differences between raw total scores 10 and 12 remain a 1-point difference in the 0–10 transformation (column D) and becomes a 6-point difference in the 0-100 transformation (column E).

Finally, and as noted in the example above, it should be pointed out that the seeming loss of information that occur when two or more adjacent ordinal raw total scores are transformed into the same linearized score should be viewed in light of the underpinning transformation and the measurement uncertainties (SEs) associated with each score. Similarly, it is noted that the choice between these transformations would not impact interpretations regarding differences at the individual person level, provided that measurement uncertainty is taken into account by using the SEs to construct 95% CIs around the transformed scores.

## Discussion

### Implications and use of logit transformations

Here we have illustrated how transformations of Rasch model derived linear logit locations into more user-friendly ranges can be conducted and interpreted in relation to their respective properties. More specifically, we have illustrated how three previously suggested transformations may be used as points of reference in evaluating the appropriateness of other user defined transformations. Arguably, the main point of transforming linear logit locations is to enhance their practical use and interpretation, since logits themselves can be confusing and difficult to interpret since they take both positive and negative values and typically are reported to the second or third decimal. This is a relevant clinical point from several perspectives [6, 7]. For example, by directly transforming ordinal raw total scores into a linear counterpart with a user-friendly range, clinicians are allowed to directly, and appropriately, use and communicate rating scale based results in an intuitive manner that allows for valid comparisons while keeping measurement uncertainty in mind. This involves communication between health care professionals as well as with patients, stakeholders and others for whom results are of relevance. As such, user-friendly transformations may represent a means of facilitating person-centred measurement and care in practice. Furthermore, the use of such linearized transformations in clinical studies will arguably improve and facilitate more valid interpretation of results, as well as provide a fundamental basis for the use of parametric statistics [2, 3]. The work presented here was conducted with the intention to ease the use of logit transformations for investigators working with quality assurance of established and development of new instruments, since procedures for such transformations are not necessarily incorporated into Rasch model estimation software. A further intention was to illustrate how the use of various transformations, in particular those based on the LMD, SEM and LSD spacing factors, can assist in associated decision-making processes. Decisions regarding the choice of transformation (or whether to transform at all) need to be made by the individual investigator. However, some general aspects to consider in this process are provided.

With established instruments where users are familiar with the raw total score range, it is likely advantageous to transform the linear logit measures into the original integer raw score range. However, in the case of a new instrument that is being developed according to the Rasch model, the developers will arguably have more options and may need to think more about what outcome range they want to establish for their new instrument. Although transformation into the original non-negative integer range still is an option, an LMD based transformation may be considered relevant as it reflects the least observable difference between scores. From the perspective of ease of score interpretation, it may be attractive to consider a transformation based on the SEM derived spacing factor since outcomes are readily interpreted under consideration of their measurement uncertainties (± 2 on the new scale will approximate the 95% CI around observed new scores). For instruments with a relatively limited raw total score range (such as the case is with the ESS illustrated above), transformations into, e.g., a 0-100 (or wider) range is not advisable for reasons outlined above. However, if the maximum raw total score approaches 100 this may well be a desirable option.

To reiterate, we suggest that the decision regarding what transformation to use should be an informed one in relation to results from the LMD and LSD based spacing factors, in addition to substantive and contextual factors, as well as the intended and expected use of the instrument. In such a process it may also be advisable to keep in mind potential further developments of, e.g., short-forms, item banks and computer adaptive testing applications, and their desirable outcome ranges relative to the original instrument.

Finally, given the need for stable calibrations from relevant well-targeted samples and acceptable model fit in the absence of threshold disordering and differential item functioning, it must be emphasized that while our example using data from the ESS is based on a fairly large sample, it is used here for illustrative purposes only and the calibrations and transformations should not be taken as final.

### An Excel tool to facilitate logit transformations

In the above example, transformations were conducted using an Excel tool, the Transforming Rasch measures in EXcel (T-REX), developed by the authors to facilitate transformations of linear logit locations based on the Rasch model. The T-REX implements the formulae summarised above, based on the work by Wright and Stone [14]. It was developed using Microsoft Excel for Microsoft 365 (version 2208, Build 16) and is freely available for download (see Additional file 1). The T-REX has been tested and found to work as intended using a range of different instruments and data sets under Windows 10, Windows 11, macOS 10 (“Catalina”), macOS 11 (“Big Sur”) and macOS 12 (“Monterey”).

The T-REX consists of three tabs, where the starting tab includes a short manual. To use the tool, the ordinal raw total scores and their corresponding linear logit person locations and logit SEs from the Rasch model estimations are needed. These values are entered in the *Scale transformation* tab together with parameters needed for the transformations: minimum and maximum logit locations, maximum possible raw total score when the minimum score is set at 0 (*L*), and desired properties for the new scale (wanted range and minimum score). In the same tab, transformed locations and their associated SEs for the new scale as well as for transformations based on the LMD, SEM and LSD spacing factors are then presented together with the spacing- and location factors used for each transformation.

In addition to transforming ordinal raw total score logit locations (as illustrated above), the tool may also be used to transform other locations on the logit scale (e.g., item- or response category threshold locations). These transformations are made in the *Locations* tab. However, since transformations of, e.g., item locations and thresholds, in general should be made into the same scale as the person locations, the parameters for transformation and properties for the new scale should first be set in the *Scale transformation* tab. Results are presented according to the same structure in both tabs.

## Conclusion

This paper has presented and discussed various approaches to transforming linear logit locations derived from the Rasch measurement model. Logits may be transformed into any range of values that preserve linearity and estimated measurement uncertainties. However, it is suggested that transformations using the previously proposed LMD, SEM and LSD based spacing factors are valuable in terms of benchmarking in deciding the new transformed scale range. We also propose an easy to use and freely available Excel tool to assist in transforming logit locations into new user defined ranges together with LMD, SEM and LSD based transformations.

## Supplementary information

**Additional file 1. **Transforming Rasch measures in EXcel (T-REX). A tool to facilitate transformations of linear logit locations based on the Rasch model.

## Data Availability

All data generated or analysed during this study are included in the paper.

## Abbreviations

CI: Confidence interval
ESS: Epworth Sleepiness Scale
LMD: The least measurable difference
Logits: Log-odds units
LSD: The least significant difference
RUMM2030: Rasch Unidimensional Measurement Model 2030
SE: Standard error
SEM: The standard error of measurement
T-REX: Transforming Rasch measures in EXcel
